# Use of Growing Cells of Pseudomonas aeruginosa for Synthesis of the Natural Vanillin via Conversion of Isoeugenol

**Published:** 2011

**Authors:** Morahem Ashengroph, Iraj Nahvi, Hamid Zarkesh-Esfahani, Fariborz Momenbeik

**Affiliations:** a *Department of Biology, Faculty of Sciences, University of Isfahan, Azadi Square, Daneshgah Street., 8174673441, Isfahan, Iran*; b *Department of Biology and Biotechnology, Faculty of Sciences, University of Kurdistan,Sanandaj, Kurdistan, I.R. Iran*.; c *Department of Chemistry, Faculty of Sciences, University of Isfahan, Isfahan 8174673441, Iran.*

**Keywords:** Biotransformation, Isoeugenol, Vanillin, *Pseudomonas aeruginosa*

## Abstract

The great demand of people for consumption of natural additives resulted in producing natural vanillin. There are plant sources and chemical procedures for vanillin production but microbial bioconversions are being sought as a suitable alternative. In the present work, the ability to produce vanillin from isoeugenol was screened using growing cultures of various bacteria. Among the 56 strains of bacteria isolated from the soil environments of Iran, a Gram-negative rod designated as strain ISPC2 showed the capability of promoting the formation of high amounts of vanillin when grown in the presence of isoeugenol. On the basis of morphological and physiochemical characteristics and 16S ribosomal ribonucleic acid (rRNA) gene sequence analysis, the isolate was identified as *Pseudomonas aeruginosa *ISPC2. Vanillin formation was analyzed by GC/FID. In the presence of isoeugenol, a growing culture of *P. aeruginosa *ISPC2 produced 1.62 g/L vanillin (molar yield of 17.3%) after a 72 h reaction at 30°C and 200 rpm. This proposed procedure is an alternative approach to obtain vanillin in an environmentally friendly way. Further studies for standardization and optimization for higher yield of vanillin production, needs to be investigated.

## Introduction

**Figure 1:**
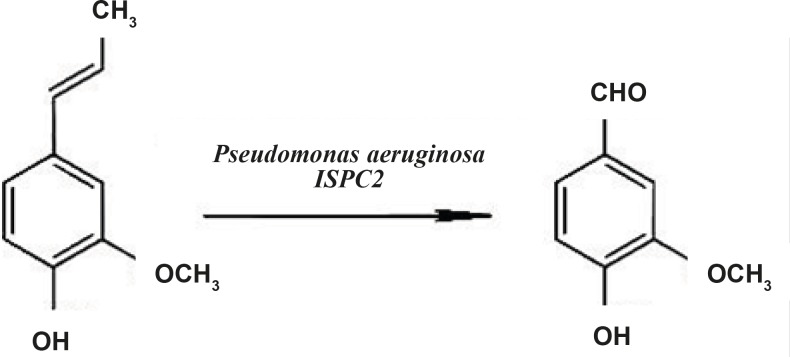
Vanillin production from isoeugenol by *Pseudomonas aeruginosa *ISPC2

Many companies in the pharmaceutical, chemical and food sectors are interested in the development of biotransformation utilizing enzymes as biocatalysts, either free, immobilized, or in whole cells (1). The products of such bioconversions are considered natural, since the European Community legislation includes products that are produced by living cells or enzymes using starting materials from a natural source under the term “natural products” (2)**. **Vanillin (C_8_H_8_O_3_, 4-hydroxy-3-methoxy benzaldehyde) is the major component of vanilla flavour extracted from the fermented pods of vanilla orchids. Vanilla pods are produced largely in Madagascar and Indonesia and contain 2-3% by weight of vanillin in the cured pod (3, 4). Preparation of natural vanillin from the vanilla pod is a laborious and slow process, which requires hand pollination of the flowers and a 1 to 6-month curing process of the harvested green vanilla pods (5). Today, only about 0.25% (40 tons out of 16,000) of vanillin sold annually originates from vanilla pods, while most of the remainder is synthesized chemically from lignin or fossil hydrocarbons, in particular guaiacol. Synthetically produced vanillin is sold for approximately US$ 15 per Kg, compared to prices of US$ 1,200 to 4,000 per Kg for natural vanillin (6). The difference between the prices combined with the increasing costumer-led demand for natural flavours has stimulated the exploration of biotechnological routes for the production of natural vanillin. The productions of vanillin based on the biocatalytic transformations of lignin, stilbenes, phenylpropanoides (*e.g. *isoeugenol, eugenol, and ferulic acid) applying fungi, bacteria, plants or genetically engineered microorganisms have been summarized previously (7-9). Isoeugenol (C_10_H_12_O_2_, 1-hydroxy-2-methoxy-4-propenylbenzene) is one of the main constituents of essential oil of clove tree and is consider a suitable precursor for a biotransformation process (8). Isoeugenol is readily obtained through isomerization of eugenol on industrial scale from cloves. Isoeugenol is chemically oxidized to vanillin using noxious reagents such as osmium tetraoxide, vanadium pentoxide or methyltrioxorhenium (5, 9). Biotransformation of isoeugenol has always been a hot topic as it is a natural renewable resource and the transformation processes are environmentally friendly. For the biotransformation of isoeugenol into vanillin, a variety of microbial species including *Aspergillus*, *Bacillus*, *Nocardia*, *Pseudomonas*, *Rdodococcus*, and *Serratia *have been tested (10-19). However, in many of the above biotransformation processes, merely trace amounts of vanillin were accumulated. The enzymatic conversion of isoeugenol to vanillin was also reported by the enzyme lipooxygenase (sigma L8383) and a crude enzyme from soybean (9, 20). In these processes, the vanillin yields from isoeugenol were 10-15%. In the present study, to find microorganisms showing high vanillin-producing activity, we carried out an extensive screening of isoeugenol-degrading microorganisms and reported for the first time the production of vanillin from isoeugenol by the newly isolated *Pseudomonas aeruginosa *ISPC2 (Figure 1).

## Experimental

*Materials and microorganisms*

Standard isoeugenol (98%, cis-trans mixture) and vanillin (99%) for biotransformation studies were obtained from Sigma-Aldrich Company. Ltd., United Kingdom. Other chemicals were of analytical grade. *Pseudomonas aeruginosa *ISPC2 and other strains were isolated from soil.

*Strain enrichment and isolation*

Different bacterial strains, including strain ISPC2, were isolated from soil samples collected from different regions of Iran. The soil samples were taken from a variety of sites as follows: an open farm planted with *Ocimum *in Kurdistan city (8 samples); a sample collected from areas beneath a clove tree in the botanical garden of Kermanshah city and 15 samples from a cultivated field on the Isfahan University farm, last planted with different types of spicy plants. The soil samples were processed for isolation of bacteria. For bacterial isolation, an enrichment culture method was used. Ten g of each wet soil sample was suspended in 90 mL of sterile distilled water and these suspensions were used as an inoculum for enrichment cultures. For the enrichment procedure, the soils were put in 25 mL of a nutrient broth (NB) medium containing 3 g bees extract, 5 g peptone and 5 g NaCl in 1 Liter of distilled water, pH 7. Isoeugenol was added to the medium at final concentrations ranging from 0.01% (v/v) to 1% (v/v). Cultures were put in a 125 mL flask containing 25 mL of medium then incubated at 30°C under agitation at 150 rpm. After five transfers, cultures were diluted and plated on nutrient agar plates. After incubation for 24-48 h at 30°C, morphologically different colonies appearing on the plates were isolated and subjected to further purification through streaking on same medium. Single colonies were picked and used for isoeugenol degradation screening.

*Primary screening of strains while transforming isoeugenol to vanillin*

To screen isolates capable of utilizing isoeugenol, cells were grown (25 mL in a 125 mL flask at 30°C, 150 rpm) on modified M9 medium (MM9), containing (in g/L): Glucose 5, (NH_4_)_2_SO_4_ 2, CaCL_2_.6H_2_O 0.32, MgSO_4_.7H_2_O 0.5, KH_2_PO_4_ 0.3, and Na2HPO_4_.12H_2_O 1.5 for 24 h, and then 1% (v/v) isoeugenol was added. Simultaneously, a control experiment was carried out by adding 1% (v/v) of isoeugenol into the sterile MM9 medium without inoculation. After an additional 48 h of incubation, potential biotransformation products were separated by acidifying (to pH of 2-3 with 10 N H_2_SO_4_) and extracted with equal volumes of ethyl acetate. The organic layer was separated by centrifugation (3,000 rpm for 1 min) and used for TLC analysis in which 10 μL samples were spotted on plates to test the degradation of isoeugenol and the accumulation of vanillin.

*Growing cultures reaction for biotransformation*

Glycerol-micronutrient-yeast extract (GMY) was designed and used in biotransformation experiments. Medium GMY comprised 10 g glycerol, 2.5 g soybean meal, 0.32 g CaCl_2_.6H_2_O, 0.5 g MgSO_4_.7H_2_O, 0.1 g KH_2_PO_4_, 3g Na_2_HPO_4_.12H_2_O, 0.153 g MnSO_4_.1H_2_O and 0.196 g CuSO_4_.5H_2_O in 1 L of distilled water with pH of 7. Biotransformation through growing cells was carried out as follows: cells were first incubated in 125 mL flasks containing a 25 mL GMY medium at 30°C in the dark with shaking at 200 rpm. After 36 h of incubation, isoeugenol was added up to a concentration of 2% (v/v). Potential products were separated by acidifying (to pH of 2-3 with 10 N H_2_SO_4_) and extracting the whole culture five times with chloroform. The organic fractions were collected, dried over anhydrous sodium sulphate, filtered, and dried by a rotary evaporator (Heidolph, Germany). The extract was then dissolved in methanol (GC grade) and analyzed by a FID gas chromatography. Simultaneously, non-inoculated cultures were performed under the same conditions to verify that isoeugenol was not partially transformed abiotically in the aerobic medium. Experiments were done in triplicate under the same testing conditions.

*Analytical methods and instrumentation*

**Figure 2:**
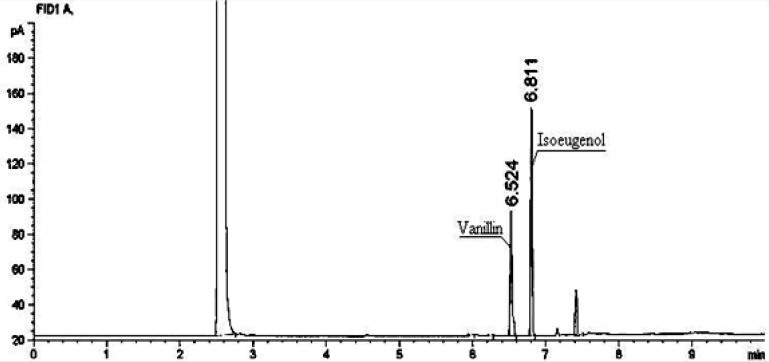
GC profile of the conversion broth showing vanillin formation from isoeugenol

TLC analysis was carried out on a 0.25 mm layer thick silica gel FG254 precoated on alumina plates [20 × 20 cm, (E.Merck, Darmstadt, Germany)]. The mobile phase was a mixture of Hexane: Ethyl acetate (3:4 v/v). Plates were dried at room temperature and visualized through UV fluorescence quenching at 254 nm under a universal transilluminator (UVp, TS-36, USA). Under these chromatographic conditions, isoeugenol and vanillin appeared and gave *R*_f_ values of 0.96 and 0.74 respectively. Vanillin was specifically detected by spraying with 2-thiobarbituric acid (0.1% solution made in 2 N HCL) which developed as a yellow-orange spot. GC analysis was performed on a flame-ionization gas chromatograph (Agilent 19091J-413) equipped with a HP-5 column (30 m × 0.32 mm × 0.25 μm film thickness). The carrier gas was He at 1 mL/min with split ratio of 50:1, injection temperature of 280°C and detection temperature of 300°C. The initial oven temperature was 80°C which was increased to 250°C at a rate of 20°C/min and then, increased at a rate of 6°C/min to the final temperature of 260°C. The system was linked to a computerized integrator. Under these conditions, retention times recorded for vanillin and isoeugenol were 6.5 min and 6.8 min, respectively (Figure 2).

*Characterization and identification of strain ISPC2*

Based on thin layer chromatography (TLC) and GC analysis, the strain giving highest vanillin yield was selected and characterized phenotypically and biochemically using standard techniques (Gram reaction, Motility, Shape and Color colony, Catalase, Oxidase, and Urease activities, nitrate reduction, Starch, Tween 20 and 80 hydrolyzes, O/F test and Indol production, *etc*.), according to the Cowan and Steel›s Manual for the Identification of Medical Bacteria (21) and Berge›s Manual of Systematic Bacteriology (22). Genomic DNA of the isolate was extracted with a Genelute DNA extraction kit (Sigma) through following the recommended procedure of the manufacturer. Sequences of the forward and reverse primers, RW01 and DG 74, used for amplification of a part of the 16S ribosomal ribonucleic acid (rRNA) gene (positions1100–1450/1500 according to the standard rRNA sequence numbering) were 5›-AACTGGAGGAAGGTGGGGAT-3› and 5›-AGGAGGTGATCCAAC CGCA-3›, respectively (23). The amplified PCR product is approximately 370 bp in length. The PCR was conducted in a 25-mL reaction volume containing 100 ng of template DNA, 1 × PCR buffer, 1 μmol/L of each universal primer (RW01 and DG74), 1.4 mmol/L MgCl_2_, 200 μmol/L of each dNTPs and 0.1 unit of Taq DNA polymerase. The sample was placed in an Eppendorf Thermal Cycler, denatured through heating for 2 min at 94°C and subjected to 30 cycles for 30 sec at 94°C, 30 sec at 60°C and 45 sec at 72°C. This was followed by a final elongation step for 2 min at 72°C. The PCR products were separated on a 1.7% agarose gel containing ethidium bromide in 1 × TBE buffer, run at 90 V for 2 h and viewed on a UV Transilluminator. The purified PCR product was sequenced in both directions using an automated sequencer by Macrogen (Seoul, Korea). The BLASTN program (http://www.ncbi.nlm.nih.gov/BLAST**) **was used for homology searches with the standard program default. Phylogenetic analysis was carried out as follows: the sequences were edited using BioEdit V.5.0.9 (24). Alignment, phylogenetic and molecular evolutionary analyses were conducted using MEGA version 4 (http://www.megasoftware.net) (25). A bootstrap test and reconstruction was done 1000 times to confirm the reliability of the phylogenetic tree (26).

## Results and Discussion

*Screening of vanillin-producing strains*

This present study is a part of a long term project for developing microbial biocatalysis in the generation of flavour and fragrance chemicals. In this work, our aim was to isolate strains capable of producing vanillin from isoeugenol. To isolate different vanillin-producing microorganisms, an enrichment culture method was used according to the protocol described in Experimental. Based on their morphologies, 56 different Isoeugenol-tolerant strains were isolated. These bacteria were then selected and tested for biotransformation of isoeugenol into vanillin. The substrate transformation was followed by TLC and GC analyses (Figure 2). Under the conditions (as mentioned under Experimental), strain ISPC2 produced the highest amount of vanillin. Based on these results, strain ISPC2 was selected for further study.

*Identification of strain ISPC2*

**Figure 3:**
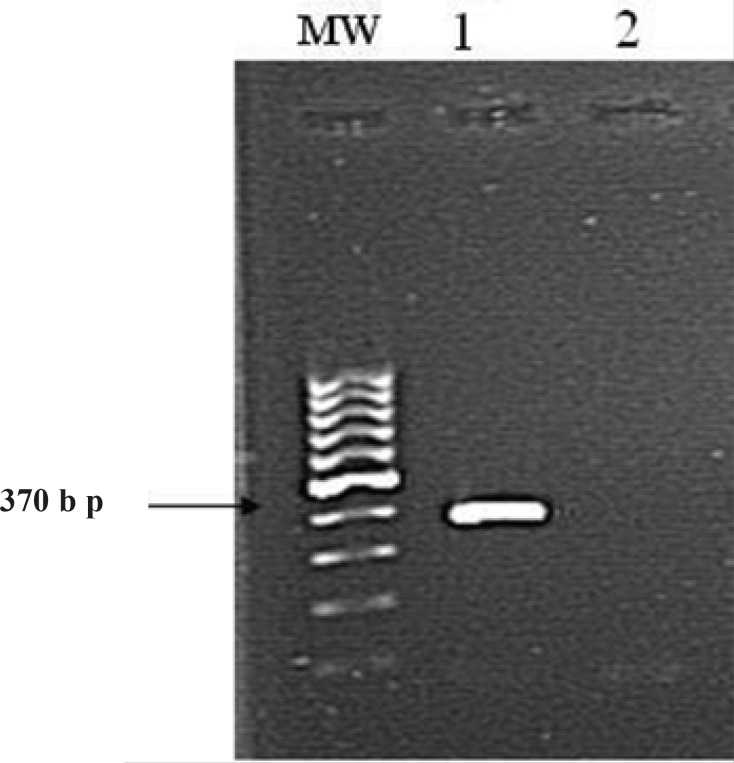
Amplification of 16S rRNA gene of isolated strain ISPC2 with universal primers RW01 and DG74

Morphological, physiological, and biochemical characteristics and 16S rRNA gene sequencing was used to identify strain ISPC2. The bacterium under investigation was isolated from soil samples collected from spicy plants farms in Isfahan, Iran. Strain ISPC2 was shown to be a Gram-negative, motile, nonspore forming, rod-shaped and produced catalase and oxidase. The colonies appeared round, smooth and formed green and brown pigments. Morphological and physiochemical characteristics of strain ISPC2 are listed in Table 1. To confirm its phylogenetic relationship with *Pseudomonas*, genomic DNA was extracted from the strain and gene coding for 16S rRNA was amplified by PCR using the universal 16S rRNA primers RW01 and DG74 (23). PCR product corresponding to the extracted size of amplified 16S rRNA (370 bp) was obtained (Figure 3). 

Lane MW, 100 bp DNA ladder; Lane 1, Strain ISPC2; Lane 2, Negative control (no added DNA).

**Figure 4:**
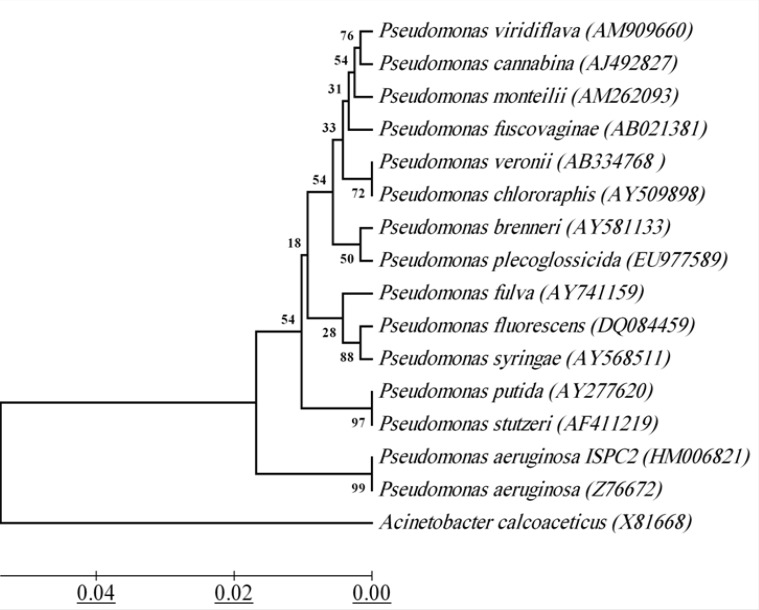
16S rRNA-based phylogenetic tree for strain ISPC2 with related species. Phylogenetic tree was constructed through Kimura 2-parameter model using the neighbor-joining method. *Acinetobacter calcoaceticus *was selected as the outgroup. Bar scale 0.02. Numbers indicate bootstrap values. GeneBank accession numbers are given in brackets

16S rRNA sequence of strain ISPC2 is submitted to the Database of GeneBank and the accession number is HM006821. The BLAST analysis showed that partial 16S rRNA of strain ISPC2 is more than 99% identical than that of *P. aeruginosa*. In order to determine the relationship between strain ISPC2 and other species of the genus *Pseudomonas *that shared highest 16S rRNA gene sequence similarities, the phylogenetic tree based on 16S rRNA was constructed (Figure 4). Based on the results of morphologic characteristics, physiological and biochemical characteristics and 16S rRNA sequence analysis, strain ISPC2 is identified as *Pseudomonas aeruginosa*.

*Results of growing cells*

**Figure 5:**
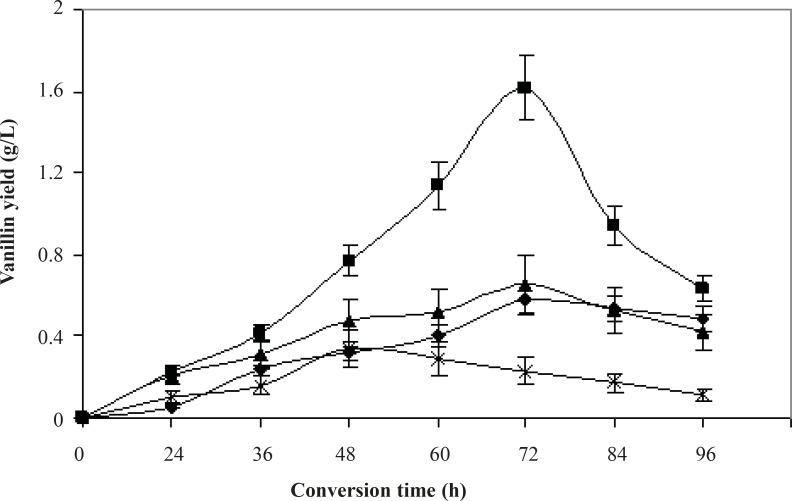
Effect of initial substrate concentration and conversion time on biotransformation of isoeugenol to vanillin through *Pseudomonas aeruginosa *ISPC2. Isoeugenol concentration (% v/v): 0.5 (**♦**), 1**(■)**, 1.5 (▲), 2 (**×**). Results represent the means of three separate experiments and deviation bars indicated

Since isoeugenol is toxic to bacterial cells, adding it directly to immature culture is unadvisable. Here, vanillin production by conversion of isoeugenol using strain ISPC2 was investigated by the addition of isoeugenol to a culture previously grown to the end of the exponential phase of growth on glycerol. Cells were first incubated in 125 mL flasks (30°C, 200 rpm) containing 25 mL GMY medium. After 36 h of incubation, the biotransformation was started by adding different concentrations of isoeugenol ranging from 0.5% to 2.0% (v/v). Samples were withdrawn at intervals of 12 h to assess the amount of formed vanillin in reaction mixture, by GC. As shown in Figure 5, vanillin production rose with the increase in the amount of substrate up to 1%. However, the concentration of vanillin did not increase further when the amount of substrate exceeded 1.5%. This may be due to the toxicity of substrate to microorganisms at its higher concentrations (substrate inhibition), as reported earlier (27), or the poor dispersibility of isoeugenol. As an aldehyde, vanillin is not stable in water and is easily oxidized to vanillic acid, which has been observed in various microbial processes (28). As shown in Figure 5, the concentration of vanillin increased at the initial stage of conversion. It began to decrease after 72 h because of the rapid oxidation of vanillin through *P. aeruginosa *ISPC2. During this biotransformation, the maximum vanillin yield of 1.62 g/L from 1% (v/v) of isoeugenol was achieved after 72 h in a 25 mL reaction mixture, resulting in a molar yield of 17.3%. The first biotransformation of isoeugenol to vanillin was achieved with *Aspergillus niger *ATCC 9142 having only 10% efficiency (10). Strains of genera *Klebsiella*, *Enterobacter *and *Serratia *were used to transform eugenol and isoeugenol to vanillin (11). 

**Table 1:** Morphological and physicochemical characteristics of strain ISPC2

**Results**	**Tests**	**Results**	**Tests**
Positive	Gelatin liquefaction	Positive	Gram reaction
Negative	H_2_S production	Rods	Shape
Positive	Indole test	Moderate	Size
Positive	Nitrite reduced	1-3 mm	Colony diameter
Oxidative	O/F test	Motile	Motility
Negative	Methyl red test	Green and Brown	Pigments
Negative	Voges-Proskauer test	Positive	Fluorescence
Positive	Arginine dihydrolase	Positive	Oxidase test
Positive	Tyrosine hydrolysis	Positive	Catalase test
Positive Positive Positive Positive	Growth on MacConkey Agar Acid production from: Glucose Fructose Glycerol	Negative Positive Positive Positive Positive Positive	Growth at temperature 4 10 20 30 37 42
Negative Negative Negative Negative Negative Negative	Lactose Maltose Sucrose Raffinose Rhamnose Cellobiose	Positive Positive Positive Positive Positive Positive Positive Positive	4 5 6 7 8 9 10 11

With *Serratia marcescence *(DSM 30126), 20.5% of isoeugenol was converted to vanillin and 3.8 g/L vanillin was obtained after 9 days. The conversion of isoeugenol to vanillin was also reported with strains of Bacillus subtilis (13) and *Pseudomonas chlororaphis *(16). Their yields were 12.4% and 12.64%, respectively. Many researches were done to improve the vanillin yield from biotransformation of isoeugenol. Cell-free extracts were used to yield 0.9 g/L of vanillin (13). Using 60% (v/v) isoeugenol as substrate, vanillin was produced at 32.5 g/L (molar yield 5.83%) over 72 h (14). A strain of *Bacillus fusiformis *has been reported for conversion of isoeugenol to vanillin, whereby product inhibition was avoided with the addition of HD-8 resin, yielding a vanillin concentration of 8.1 g/L from 50 g/L isoeugenol (29). Recently, by using resting cells of *Pseudomonas putida *IE27, vanillin was produced at 16.1 g/L (molar yield 71%) after 24 h of incubation in the presence of 10% (v/v) DMSO (18). Further studies for improving the yield of vanillin using *Pseudomonas aeruginosa *ISPC2 are under progress. Natural flavour and fragrance chemicals are in many cases highly expensive due to their limited availability from natural sources. In fact, most of the flavour and fragrance chemicals are produced by chemical synthesis. Due to the increasing demand for healthy and natural food, there is a considerable interest in utilizing microbial transformation to produce vanillin from natural materials by biotransformation, which can then regarded as a natural aroma chemical. As one of the most important components of natural flavours, vanillin is widely used in foods, beverages, perfumes, pharmaceuticals, and medical industries (8). In the present work, we investigated vanillin production through microbial transformation using isoeugenol as a precursor. Isoeugenol, which is a commercially available natural raw material with a market prize of US$ 9 per Kg, will be an excellent starting material for microbial conversion to vanillin. Isoeugenol is usually obtained from eugenol by simple isomerization and it can also be extracted from the clove tree *Syzygium aromaticum *directly. Eugenol and isoeugenol account for more than 90% of the essential oil extracted from cloves. During a screening for microorganisms which were able to convert isoeugenol to vanillin, a novel strain, *Pseudomonas aeruginosa *ISPC2, was isolated from soil samples collected from spicy plants farms. Based on physiological and biochemical description as well as partial 16S rRNA sequencing, strain ISPC2 was identified as *P. aeruginosa*. Although other *Pseudomonas *sp. strains (*Pseudomonas putida*, *Pseudomonas chlororaphis*) can convert isoeugenol to vanillin, the present study has provided the first evidence for conversion of isoeugenol to vanillin through *P. aeruginosa *ISPC2. Using 1% (v/v) of isoeugenol as substrate, 1.62 g/L vanillin was produced in a 25 mL reaction solution after 72 h, at 30°C and 200 rpm. However, production of vanillin from isoeugenol through this biotechnological route is not very economical as the vanillin level was 1.62 g/L at the end of 72 h. Further studies in this direction for obtaining higher yields of vanillin are in progress.
